# Clonal Hematopoiesis with Oncogenic Potential (CHOP): Separation from CHIP and Roads to AML

**DOI:** 10.3390/ijms20030789

**Published:** 2019-02-12

**Authors:** Peter Valent, Wolfgang Kern, Gregor Hoermann, Jelena D. Milosevic Feenstra, Karl Sotlar, Michael Pfeilstöcker, Ulrich Germing, Wolfgang R. Sperr, Andreas Reiter, Dominik Wolf, Michel Arock, Torsten Haferlach, Hans-Peter Horny

**Affiliations:** 1 Department of Internal Medicine I, Division of Hematology & Hemostaseology, Medical University of Vienna, 1090 Vienna, Austria; wolfgang.r.sperr@meduniwien.ac.at; 2 Ludwig Boltzmann Institute for Hematology & Oncology, Medical University of Vienna, 1090 Vienna, Austria; gregor.hoermann@meduniwien.ac.at (G.H.); Jelena.Milosevic-Feenstra@onc.lbg.ac.at (J.D.M.F.); michael.pfeilstoecker@wgkk.at (M.P.); 3 MLL Munich Leukemia Laboratory, 81377 Munich, Germany; wolfgang.kern@mll.com (W.K.); torsten.haferlach@mll.com (T.H.); 4 Central Institute of Medical and Chemical Laboratory Diagnostics, University Hospital Innsbruck, 6020 Innsbruck, Austria; 5 Department of Laboratory Medicine, Medical University of Vienna, 1090 Vienna, Austria; 6 Institute of Pathology, Paracelsus Medical University, zip code Salzburg, Austria; k.sotlar@salk.at; 7 3rd Medical Department, Hanusch Hospital, 1140 Vienna, Austria; 8 Department of Hematology, Oncology, and Clinical Immunology, Heinrich-Heine-University, 40204 Düsseldorf, Germany; Germing@med.uni-duesseldorf.de; 9 Department of Hematology and Oncology, University Medical Centre Mannheim, University of Heidelberg, 68167 Mannheim, Germany; Andreas.Reiter@medma.uni-heidelberg.de; 10 Department of Hematology and Oncology, Medical University Innsbruck, 6020 Innsbruck, Austria; dominik.wolf@i-med.ac.at; 11 Medical Clinic 3, University Clinic Bonn (UKB Bonn), 53127 Bonn, Germany; 12 Laboratory of Hematology, Pitié-Salpêtrière Hospital, 75013 Paris, France; michel.arock@aphp.fr; 13 Institute of Pathology, Ludwig-Maximilians University, 80539 Munich, Germany; Hans-Peter.Horny@med.uni-muenchen.de

**Keywords:** premalignant states, neoplastic stem cells, clonal evolution, cancer

## Abstract

The development of leukemia is a step-wise process that is associated with molecular diversification and clonal selection of neoplastic stem cells. Depending on the number and combinations of lesions, one or more sub-clones expand/s after a variable latency period. Initial stages may develop early in life or later in adulthood and include premalignant (indolent) stages and the malignant phase, defined by an acute leukemia. We recently proposed a cancer model in which the earliest somatic lesions are often age-related early mutations detectable in apparently healthy individuals and where additional oncogenic mutations will lead to the development of an overt neoplasm that is usually a preleukemic condition such as a myelodysplastic syndrome. These neoplasms may or may not transform to overt acute leukemia over time. Thus, depending on the type and number of somatic mutations, clonal hematopoiesis (CH) can be divided into CH with indeterminate potential (CHIP) and CH with oncogenic potential (CHOP). Whereas CHIP mutations *per se* usually create the molecular background of a neoplastic process, CHOP mutations are disease-related or even disease-specific lesions that trigger differentiation and/or proliferation of neoplastic cells. Over time, the acquisition of additional oncogenic events converts preleukemic neoplasms into secondary acute myeloid leukemia (sAML). In the present article, recent developments in the field are discussed with a focus on CHOP mutations that lead to distinct myeloid neoplasms, their role in disease evolution, and the impact of additional lesions that can drive a preleukemic neoplasm into sAML.

## 1. Introduction

It is generally appreciated that cancer evolution is a step-wise process that is associated with molecular diversification and clonal selection of neoplastic stem cells [1,2,3,4,5,6]. Blood cancer evolution may begin early in life or later in adulthood and includes premalignant and malignant stages. Thus, in many instances, the development of (blood) cancer is a long-lasting process that takes several years or even decades [1,2,3,4,5,6,7,8]. In chronic (indolent) myeloid neoplasms, such as the myelodysplastic syndromes (MDS), myeloproliferative neoplasms (MPN), or chronic myeloid leukemia (CML), the disease is separable into premalignant (non-aggressive) phases and a terminal phase that resembles secondary acute myeloid leukemia (sAML) [9,10,11,12,13,14,15]. However, even before an overt myeloid neoplasm, such as a MDS, develops, a clonal pre-phase of the disease may be detected by chance or by screening apparently healthy individuals [8,16,17,18,19,20,21,22]. Such pre-phase is defined by normal or near normal blood counts and detection of one or more somatic mutations, which *per se* (as isolated lesion) exhibit low oncogenic potential [8,16,17,18,19,20,21,22]. Whereas, in some of these individuals, a myeloid neoplasm develops in the follow-up, others will never develop an overt myeloid disorder during their lifetime. The risk of disease evolution (risk to develop a myeloid neoplasm) depends on the type of mutations and other factors. Clonal hematopoiesis (CH) and the related mutations can thus be divided into CH with indeterminate potential (CHIP) and CH with oncogenic potential (CHOP) [8].

**Figure 1:**
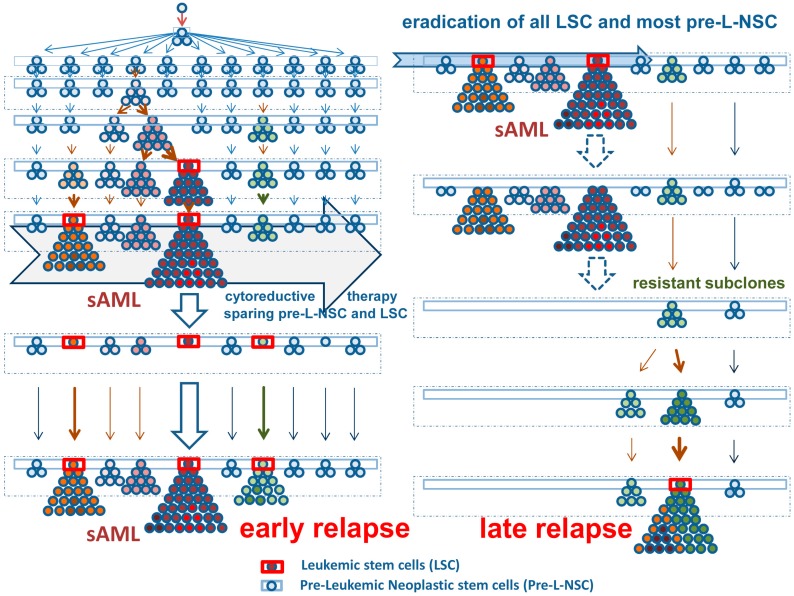
Development and diversification of leukemic stem cells in secondary acute myeloid leukemia (sAML). Left panel, upper part: An initial oncogenic event transforms a normal stem cell into a premalignant (preleukemic) neoplastic stem cell (Pre-L-NSC) (blue boxes). These cells or their daughter cells acquire early somatic mutations. Usually, these have low oncogenic potential (blue-colored cells) and are thus slowly cycling or dormant cells so that the mutation is not detectable. After some time, more daughter clones develop and the somatic lesions may be detected and classified as clonal hematopoiesis with indeterminate potential (CHIP). After several years or decades, one or more daughter clones and their stem cells expand and may replace normal hematopoiesis. At that time, some of the stem cell clones may have acquired disease-specific oncogenic driver lesions (red-colored cells). Still, these cells may be indistinguishable from normal cells by morphology and in functional terms. In a next step, one or more of the sub-clones acquire additional driver mutations or lose tumor suppressor genes. As a result, the stem cells are now cycling and the neoplastic process forms a visible overt myeloid neoplasm (red-colored prominent clones—upper left panel). In most instances, these neoplasms still behave as indolent driver-positive neoplasm for some time. However, unless treated, many of these conditions will finally transform into a secondary acute myeloid leukemia (sAML). At that time, long-term disease propagating cells are called leukemic stem cells (LSC—red boxes). Note that all of the Pre-L-NSC-derived clones are also still present and can be detected (as small-sized sub-clones) in an overt sAML. Left lower panel: Nonspecific cytoreductive (palliative) therapy (example: hydroxyurea) can suppress the growth of cycling stem and progenitor cells for some time but cannot eradicate any of the Pre-L-NSC or LSC. After a variable (usually short) time period, a relapse develops. Right panel: Most interventional therapies (intensive chemotherapy, targeted drugs, or stem cell transplantation) are able to eradicate most or all of the LSC and their progeny, but not all Pre-L-NSC. When all LSC are killed, the patient enters complete remission and operational cure. In these patients, the Pre-L-NSC may or may not be detected as minimal residual disease. These Pre-L-NSC may (or may not) produce a late relapse after several months or years. Although some of the early mutations (rarely even drivers) of the original sAML clone may be detected in such relapsing disease, the molecular aberration profiles usually differ substantially from the initial molecular make-up of the sAML clone.

We and others have recently proposed a model of cancer evolution where the earliest stages of carcinogenesis are defined by expression of somatic mutations in small-sized (yet stem cell-containing) cell fractions [8,19,20,21,22,23,24,25]. Later, over time, one or more sub-clone(s) expand(s) and replace normal cells, depending on additional somatic lesions (Figure 1) [8]. As long as the neoplastic cells retain their full differentiation and maturation potential and can be controlled by the niche and the immune system, the involved clones will remain indolent and may, over time, replace or even mimic (at least to a degree) the normal organ in functional and morphological terms. However, as soon as the dominant clone(s) escape(s) most control mechanisms, the disease will further expand and progress into an aggressive malignancy. The end-stage of such malignancy (sAML) is usually resistant not only against most endogenous control systems, but also against diverse therapeutic maneuvers [9,10,11,12,13,14,15].

Depending on the disease variant and the types and numbers (combinations) of somatic mutations acquired, the premalignant phases of a myeloid neoplasm bear a low or high risk to transform to sAML [8,19,20,21,22,23,24,25]. Interestingly, certain myeloid neoplasms, such as advanced MDS, most MDS/MPN overlap disorders, and chronic phase CML bear a (relatively) high risk, whereas other clonal conditions are at a clearly lower risk, but may eventually also transform to sAML. These include, among others, chronic eosinophilic leukemia (CEL), MDS with isolated del(5q), or indolent systemic mastocytosis (ISM). So far, it remains unknown why some conditions are associated with a relatively high risk of sAML transformation. Possible explanations are the differential oncogenic potential of various driver mutations and the presence of additional germline or somatic mutations.

In the current article, we review the mutational landscape of myeloid neoplasms and try to link individual mutations and mutation-combinations to clinical phenotypes and the risk of progression. We also discuss why CHOP mutations can trigger disease evolution and why some CHOP mutants are less oncogenic than others.

## 2. Clonal Hematopoiesis (CH) of Indeterminate Potential (CHIP)

The term CHIP was coined to describe the presence of clonal somatic mutations (otherwise detected in myeloid neoplasms: MDS, AML, and others) in leukocytes obtained from apparently healthy individuals or subjects with minimal blood count abnormalities [8,21,22,26,27,28]. In patients with CHIP, diagnostic criteria for MDS, MPN, or other myeloid neoplasms are not fulfilled even if the size of the ’CHIP clone’ is substantial. Most patients with CHIP are older healthy individuals. Therefore, the term age-related clonal hematopoiesis (ARCH) was also proposed [19]. In patients with CHIP, the risk to develop a myeloid (hematopoietic) neoplasm is slightly elevated compared to controls without CH [18,19,20,21,26,27,28]. In addition, these patients may be at relatively high risk to develop progressive atherosclerosis and related cardiovascular disorders [19,29]. In a subset of patients with CHIP/ARCH, however, no malignancy and no severe cardiovascular disease develop.

In some individuals, the CHIP clones are small-sized and may thus escape detection by conventional screening/sequencing approaches. However, most next generation sequencing (NGS) assays have sufficient sensitivity to detect relatively small clones (mutant allele burden 1–5%) and thus represent the preferred method for the diagnostic assessment of CHIP. NGS assays can also be modified to reliably detect even smaller clones (mutant allele burden clearly <1%). These very small hematopoietic cell clones are currently not considered as CHIP per definition since their prevalence is even higher and their clinical impact remains unclear. The generally accepted definition of CHIP includes a minimal allele burden of 2%, the absence of persistent (≥4 months) cytopenia and exclusion of an underlying overt pathology associated with the somatic lesion [21,22]. The term CHIP should thus only be applied to individuals who have normal blood counts. In the case that slight cytopenia is also detected and the criteria for MDS or other myeloid neoplasms are not fulfilled, the diagnosis changes to clonal cytopenia of undetermined potential (CCUS), which is a rare condition [21,22]. When detected as an isolated defect (in the absence of other lesions or loss of tumor suppressors), CHIP mutations are indicative of a rather good prognosis regarding clonal stability, and only a small subset of these individuals will eventually develop a hematopoietic neoplasm over time [18,19,20,21]. A list of frequently reported CHIP mutations is provided in Table 1.

CHIP-like mutations can also be detected in patients with overt myeloid neoplasms, including MDS, AML, and mast cell neoplasms (Table 1 and Table 2) [30,31,32,33,34,35,36,37,38,39]. However, although some of these mutations are more frequently detected in certain myeloid neoplasms than others, most are not disease-specific. Another interesting aspect is that CHIP-like mutations can sometimes also be detected after successful therapy when the predominant clones have been eradicated [40,41,42,43]. This is best explained by the stem model of cancer evolution where early sub-clones are formed and acquire these mutations, but only a few of these sub-clones expand after acquiring driver lesions and additional mutations over time (Figure 1) [8,23,44,45,46,47,48]. After debulking, the early sub-clones may still be present because of their slow proliferation rate and drug resistance (Figure 1). Such early clones exhibiting CHIP-like lesions may either remain silent for the lifetime of the host or they become relevant clinically: First, they may produce a late and usually driver-negative relapse (Figure 1). Second, they may contribute to the risk to develop a severe cardiovascular (vascular occlusive) disease [19,29,40,41,42,43].

In myeloid neoplasm, isolated CHIP-like mutations may still be indicative of a rather good prognosis regarding clonal stability [30,31,32,33,34,35,36,37,38,39]. However, in the context of an overt myeloid neoplasm, CHIP-like mutations are often indicative of a poor prognosis, especially when multiple CHIP-like mutations are expressed, or the CHIP-like mutation is accompanied by clonal hematopoiesis of oncogenic potential or loss of a tumor suppressor gene (Table 2) [30,31,32,33,34,35,36,37,38,39].

It was also described that the prognostic impact of CHIP-like mutations regarding survival and AML evolution depends on (i) the type of the mutation, (ii) the dynamics of clonal evolution (rapid expansion of sub-clones carrying CHIP-like mutations is a poor prognostic sign), and (iii) the underlying primary neoplasm. For example, a stable *TET2* mutation in low-risk MDS may be indicative of a good prognosis, whereas a rapidly expanding *ASXL1*-mutated clone in CMML-2 has to be regarded as a poor prognostic sign concerning AML evolution.

## 3. Clonal Hematopoiesis of Oncogenic Potential (CHOP) and Separation from CHIP

Whereas isolated CHIP-type mutations can be detected in healthy individuals who stay healthy for their lifetime, CHOP mutations are usually associated with manifestation of an overt neoplasm. In fact, most of these individuals will develop a hematopoietic malignancy, although CHOP-positive cases presenting with a long-lasting disease-free survival have been described. For example, although *BCR-ABL1* can be detected in small-sized clones in a few healthy individuals [16], most patients with a persistent *BCR-ABL1*+ clone have or will develop a *BCR-ABL1*-positive leukemia. Even in those with low mutant burden, *BCR-ABL1* must be regarded as a high-risk condition (CHOP) that is likely to transform to an overt leukemia after a variable latency period. As in other myeloid neoplasms, the CML clone acquires additional lesions and hits when the disease progresses into accelerated phase and blast phase [64,65,66]. In addition, early CHIP-like lesions may be detected in the CML clone [64,65,66]. These lesions may also persist during successful treatment with BCR-ABL1 tyrosine kinase inhibitors (TKI) and may confer a potential risk for the development of cardiovascular events during treatment with certain TKI, such as nilotinib [67].

In the *JAK2*-mutated MPN, the situation is similar compared to CML. However, in contrast to *BCR-ABL1*, the *JAK2* V617F mutation status *per se* confers a high risk for the development of cardio-vascular events [17,68,69,70,71]. Therefore, *JAK2* V617F+ hematopoiesis is often detected quite early, sometimes long before an overt MPN is diagnosed. However, again, the risk for the development of an MPN is high and most patients will eventually develop such a disease over time [17,68,69,70,71]. Therefore, although *JAK2* V617F exhibits some features of a CHIP-like mutation, it is generally considered to belong to the group of CHOP mutations.

In patients with MPN, other driver lesions may also be detected, including mutations in the calreticulin (*CALR*) gene or in the *MPL* gene [72,73,74]. In addition, apart from these drivers and *JAK2* V617F, a number of additional mutations may be detected in patients with MPN. These include, among others, mutations in *TET2*, *ASXL1*, *SRSF2*, *DNMT3A*, *U2AF1*, *CBL*, *KIT*, *RUNX1*, and *EZH2* [13,75,76] (Table 1 and Table 2). Moreover, neoplastic cells in MPN patients may acquire mutations in *TP53*, which is usually associated with disease progression to sAML [13,75,76].

In MDS, no classical recurrent driver lesions, such as *BCR-ABL1* or *JAK2* V617F, have been identified. Rather, in these patients, a number of different molecular lesions and combinations of somatic mutations are found [30,31,32,33,34,35]. Based on clinical correlates regarding progression and survival, these lesions may be divided into CHIP-like mutations (Table 1 and Table 2) and more oncogenic (CHOP-like) driver mutations (Table 2 and Table 3). The latter are associated with a substantial risk to transform to AML, and include, among other, mutations in *FLT3*, *RUNX1*, *WT1*, *NPM1*, *NRAS*, and *TP53* [30,31,32,33,34,35,77]. However, these CHOP mutations are usually not detected in a pre-diagnostic phase (in healthy subjects) but only (mostly) in the context of a full-blown MDS or AML.

In chronic myelomonocytic leukemia (CMML), a classical MPN/MDS overlap disorder, mutations can also be divided into CHIP-like and more oncogenic (CHOP-like) mutations (Table 2). Genetic lesions that are typically detectable in CMML include mutations in *SRSF2* (about 50% of patients), *TET2* (50–60%), and *ASXL1* (35–49%) (Table 1). Mutations associated with disease progression to sAML include mutations in *CBL*, *NRAS, KRAS*, *RUNX1,* and *SETBP1* [55,78,79,80,81,82]. These mutations are detectable in at least 10% of all patients with CMML and are associated with poor prognoses. Especially RAS-pathway mutations and complex mutation patterns are highly predictive for progression to sAML. Mutations in *ASXL1* and *TET2* are also detected frequently in CMML. As an isolated lesion in CMML, a *TET2* mutation may be regarded as a CHIP-like mutation. However, in the CMML-context, *ASXL1* mutations are indicative of a poor prognosis, especially when other mutations are also expressed in CMML cells [83,84,85]. The same holds true for other rare driver mutations, such as *BRAF* mutations or *FLT3* mutations [86,87]. An overview of CHOP-type mutations is provided in Table 2.

In *de novo* AML, a number of disease-related or even AML-specific driver mutations, such as *RUNX1-RUNX1T1* (*CBFB-MYH11*), *PML-RARa*, *FLT3* mutations, *KIT* D816V, or *NPM1* mutations, have been identified [37,88,89,90,91,92]. Some of these mutations are even diagnostic and serve as diagnostic AML criteria in the World Health Organization (WHO) classification [37,89,90]. Other mutations contribute to clonal expansion of leukemic cells and are indicative of a poor prognosis (examples: *TP53* mutations, RAS pathway mutations, multiple somatic mutations) similar to the situation in CMML [37,89,90]. In addition, CHIP-like mutations can be detected in both *de novo* AML patients and (even more frequently) in patients with sAML [37,89,90,91].

Based on preclinical data and clinical studies, the low oncogenic potential of individual CHIP-like mutations may change to a higher oncogenic potential when the lesion is expressed together with other CHIP-like mutations or with a CHOP-type driver lesion. Indeed, in most disease models analyzed, the presence of multimutated sub-clones is usually associated with disease progression and an unfavorable outcome [36,37,38,39,82,83,84,85,89,90]. An exception may be the presence of two CHIP-like mutations in two coexisting and clearly separable (independent) sub-clones.

Another important aspect is that oncogenic (CHOP-like) mutations may also be expressed in the germline, thereby leading to a familial predisposition to develop a myeloid neoplasm, including AML. Such germline mutations were described in *RUNX1*, *CBL, KIT*, and other target genes [92,93,94,95]. In addition, it was described that certain gene constellations (genetic patterns or gene variations) predispose for the acquisition of CHOP mutations, such as *JAK2* V617F (MPN) or *KIT* D816V (mastocytosis) [96,97,98,99]. In these conditions, familial clustering of MPN or mastocytosis is found, but the oncogenic driver lesion is a somatic defect [96,97,98,99].

Finally, inherited gene defects may be associated with loss of tumor suppressor genes or tumor suppressor function and may thereby contribute to the development of myeloid neoplasms, including MDS, CMML, and AML.

## 4. Classification of CHOP: Drivers of Differentiation, Proliferation, Maturation, and/or Oncogenesis

The term ‘oncogenic’ is often used to describe a somatic process that converts a non-neoplastic or a premalignant neoplastic condition into an overt malignancy. This definition implies that these mutations (or other events) provide a growth and survival advantage over normal cells. However, in many instances, the driver mutation *per se* is promoting differentiation and maturation rather than proliferation, unless additional mutations are also expressed by neoplastic cells.

In general, CHOP mutations can be divided into (a) disease specific drivers that trigger differentiation and maturation without promoting substantial proliferation (weak oncogenes) like *KIT* D816V, (b) disease-specific drivers of differentiation and proliferation of hematopoietic (stem) cells, like *BCR-ABL1*, and (c) drivers that preferentially trigger the proliferation of hematopoietic stem and progenitor cells but do not or only marginally promote differentiation, such as mutant forms of *RAS*. In most instances, tumor suppressor loss (functionally by mutations or loss of genetic material) is also associated with enhanced proliferation of neoplastic (stem) cells.

The Ba/F3 model with doxycycline-inducible expression of oncogenes is a useful tool to define the differentiation and proliferation capacity of disease-related drivers. For example, expression of *KIT* D816V induces histamine production and (mast cell) differentiation, but not proliferation in Ba/F3 cells, whereas expression of *BCR-ABL1* induces both histamine production and proliferation in Ba/F3 cells [100]. Correspondingly, patients with indolent systemic mastocytosis, where *KIT* D816V is usually the only driver lesion, accumulate their mast cell aggregates over years or even decades, and have a stable disease course with normal life expectancy. In these patients, most neoplastic mast cells are mature cells that do not proliferate and even the immature mast cell progenitor cells show no enhanced proliferative capacity over normal cells (the total burden of mast cells remains stable) unless additional mutations are acquired. By contrast, in *BCR-ABL1*+ chronic phase CML, uncontrolled proliferation of myelopoietic cells is a key feature, and when untreated, the disease rapidly transforms to accelerated phase and blast phase with short survival times.

Another important aspect is that some of the oncogenic driver mutants, such as BCR-ABL1 or JAK2 V617F, are *per se* capable of promoting clonal instability. For example, it was described that BCR-ABL1 promotes the acquisition of secondary mutations by a pathway involving STAT5 and increased production of reactive oxygen species (ROS), with consecutive DNA damage and clonal instability [101]. Similarly, JAK2 V617F was described to induce clonal instability and loss of heterozygosity via mutant-induced generation of ROS [102].

These additional lesions and hits are required for leukemogenesis and are indeed found in high-risk patients and those who actually progress to sAML. Sometimes, the primary driver may even suppress evolution to sAML because of its differentiation-inducing (and thus proliferation-hindering) effects. Therefore, it is not unexpected that in patients with ISM or MPN who progress to sAML, the AML clone often becomes or is negative for the molecular driver lesion. Especially when these patients are treated with drugs directed against these drivers, progression to sAML is often accompanied by a ‘loss’ of the driver, which can be explained by the selection of more malignant, ‘initial driver-negative’ sub-clones. A summary of CHOP mutations and secondary driver lesions and their functional impact in oncogenesis in myeloid neoplasms is provided in Table 2.

## 5. CHIP and CHOP in the Context of Leukemic Stem Cells

The concept of leukemic stem cells (LSC) has been coined to explain cellular hierarchies, sub-clone formation, and the directed diversification of clonal cell populations in diverse malignancies [44,45,46,47,48,103,104]. In addition, the LSC concept was propagated with the idea to focus research work and translational approaches on disease-initiating and -propagating cells. In fact, it is clear that antineoplastic therapies can only be curative in nature when eliminating most or all neoplastic stem cells fractions in a given malignancy [103,104,105].

**Figure 2:**
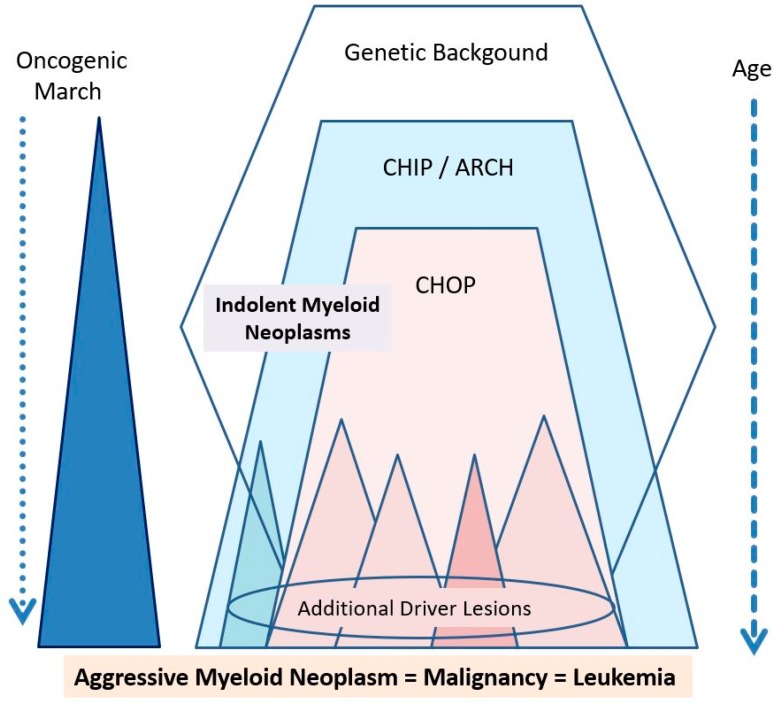
Major players contributing to the ‘oncogenic march’ in myeloid neoplasms. The genetic background may form the basis of a familiar predisposition to the development of hematopoietic (and thus also myeloid) neoplasms. In some of these families, more or less specific of even disease-related mutations (with low or even high oncogenic potential) are found. CHIP develops later during lifetime—the related somatic mutations *per se* (as isolated lesions) have a low oncogenic potential and are more frequently detectable at higher age. Therefore, these lesions are also called age-related clonal hematopoiesis (ARCH). Later, somatic mutations with CHOP may be acquired and usually lead to an overt myeloid neoplasm (at least after some time). This neoplasm may manifest as an indolent (chronic) myeloid neoplasm unless additional drivers (driver lesions) and other oncogenic hits (loss of tumor suppressors) are acquired. In a few cases, such additional driver lesions may be acquired in a CHIP status (blue triangle) or even a pre-CHIP status and may then lead to the immediate formation of primary (*de novo*) AML.

In the past few years, neoplastic stem cells have been classified into premalignant (preleukemic) neoplastic stem cells (pre-L-NSC) and leukemic (malignant) neoplastic stem cells (LSC), also termed cancer stem cells in the context of a solid cancer [45,46,47,48]. Pre-L-NSC give rise to small-sized clones and usually behave very similar compared to normal hematopoietic stem cells. By contrast, LSC give rise to an overt leukemia or another advanced blood cell cancer. It is generally accepted that at least one or more CHIP mutations are required to convert a normal (stem) cell into a Pre-L-NSC. CHOP mutations may also be expressed in Pre-L-NSC, especially in chronic myeloid neoplasms. However, in these patients, Pre-L-NSC may convert into LSC within short time. Based on the model of step-wise evolution of cancer/leukemic stem cells (Figure 1), LSC and their progeny (= leukemic cells) must be expected to contain a mixture of CHIP and CHOP mutations. However, in rare cases, CHOP-negative sub-clones expand, especially under targeted therapy (Figure 1 and Figure 2). In these cases, other oncogenic mutant forms may be expressed or the loss of certain tumor suppressors may introduce an additional oncogenic player.

All in all, the CHIP vs. CHOP concept is in line with and nicely explains the classification of NSC into Pre-L-NSC and fully malignant/leukemic NSC = LSC. Whereas Pre-L-NSC are more likely to contain one CHIP mutation, LSC are more likely to express two or more CHIP mutations or (more frequently) at least one CHOP mutation.

An important aspect, when considering potentially curative concepts, is LSC resistance. In fact, LSC are known to exhibit multiple forms of drug resistance, including intrinsic stem cell resistance (natural defense against toxins), acquired drug resistance (often mediated by secondary mutations in driver target genes or mutations in additional genes), niche-mediated resistance, and immune-mediated resistance (often triggered by checkpoint molecules). In addition, pharmacologic resistance may occur. Finally, LSC sub-populations may confer resistance because of the lack of major drug targets [45,46].

## 6. CHOP-Mutant Forms as Major Targets of Therapy

Because of their specificity and obvious function as major drivers of oncogenesis in various myeloid neoplasms, CHOP mutant forms were recognized as major targets of therapy. It started with the notion that BCR-ABL1-targeting TKI, such as imatinib, could effectively suppress growth and survival of leukemic cells in patients with Ph+ CML [106]. Later, imatinib was also found to block the kinase activity of FIP1L1-PDGFRA, the major driver of oncogenesis in chronic eosinophilic leukemia (CEL) and in a subset of related myeloid malignancies [107,108]. Subsequently, a number of different driver mutants and drugs directed against these drivers have been examined. In the classical MPN, inhibitors of JAK2 V617F were applied with considerable success [109]. In advanced systemic mastocytosis, drugs targeting the oncogenic KIT D816V mutant have been developed [110,111]. Finally, in AML, drugs directed against FLT3 ITD, IDH2 mutants and other oncogenic mutants were developed and are applied together with poly-chemotherapy in these patients.

All in all, targeting of CHOP-like mutant forms seems to be an effective approach in many patients. However, not all patients respond or show long lasting remissions. Rather, neoplastic cells are often resistant or develop resistance against these drugs. A number of different mechanisms of resistance against driver mutants have been deciphered in recent years. A full description and review of these mechanisms is beyond the scope of this article. The reader is referred to the available literature. In general, fusion genes encoding for CHOP-like drivers can acquire secondary mutations through which drug resistance develops. Second, driver-negative sub-clones can emerge. Third, niche-related and immunological forms of resistance may contribute to overall drug resistance. Finally, intrinsic stem cell resistance and pharmacological resistance may occur [45,46]. All these forms of resistance can be found in myeloid neoplasms and are often critically involved in MDS, CMML, and AML.

A logical way to overcome the multiple forms of resistance against CHOP driver-directed drugs is the application of drug combinations. Such combinations are currently being tested in preclinical and clinical studies.

## 7. Concluding Remarks and Future Perspectives

The term CHOP was proposed for somatic mutations that drive oncogenesis in various hematopoietic neoplasms as a single hit or cooperative hit that acts pro-oncogenic in somatic aberration networks. Whereas some of these drivers may directly induce the proliferation of neoplastic stem and progenitor cells, others induce differentiation in distinct hematopoietic lineages and are therefore disease-specific and lineage-related and often detected in premalignant chronic neoplasms. Some of these drivers may *per se* promote oncogenesis through the induction of clonal instability. Finally, in the context of multimutated sub-clones, oncogenic drivers contribute to the transformation to sAML. CHOP-related mutants have also been recognized as promising targets of therapy in myeloid neoplasms. However, complete suppression of oncogenesis and eradication to cure require the elimination of all premalignant and malignant neoplastic stem cells.

## Figures and Tables

**Table 1:** Examples of mutations that have been described in the context of clonal hematopoiesis of indeterminate potential (CHIP) or age-related clonal hematopoiesis (ARCH).

	Reported Frequency (% of Cases) in Patients with					
Mutated Gene	CHIP	MDS	CMML	MPN	AML	AdvSM
*DNMT3A*	50–60	5–15	1–10	1–12 *	15–35	5–15
*TET2*	10–15	20–30	50–60	18–45 *	<1–10 **	30–40
*ASXL1*	8–10	15–20	35–40	5–35 *	1–10 **	15–20
*SF3B1*	2–5	20–30 ***	5–10	5–10	<1–10	<1
*GNB1*	3–4	<1	<1	<1	<1	<1
*SRSF2*	1–2	15–17	45–50	<1–18 *	5–10	35–40
*GNAS*	1–2	<1	<1	<1	<1	<1

* The broad range in patients with myeloproliferative neoplasms (MPN) is due to a variable distribution of these mutations among the three major entities: polycythemia vera (PV), essential thrombocythemia (ET), and primary myelofibrosis (PMF—where these mutations occur more frequently). ** The broad range is due to a different prevalence of these mutations in various AML categories. In general, these mutations are more frequently detected in secondary AML, following MDS or CMML. *** The mutant *SF3B1* status is associated with deletions in the long arm of chromosome 11 and with the presence of ring sideroblasts in MDS. Abbreviations: MDS, myelodysplastic syndromes; CMML, chronic myelomonocytic leukemia; MPN, myeloproliferative neoplasms; AML, acute myeloid leukemia; AdvSM, advanced systemic mastocytosis.

**Table 2:** Some clinically relevant somatic mutations detectable in myeloid neoplasms based on oncogenic potential and risk to transform to secondary acute myeloid leukemia (sAML).

Myeloid Neoplasm	Clinically Relevant Somatic Mutations		
	Early (CHIP-Like)	Specific/Driver	Late/Transforming
MDS [49,50,51,52]	*TET2*, *DNMT3A*, *IDH1/2* *	*SF3B1*, *SRSF2*, *U2AF1*, *ZRSR2* **	*ASXL1* ***, *RUNX1*, *TP53*, *EZH2*, *SETBP1*, *STAG2*, *NPM1*, *FLT3*, *PTPN11*, *N/KRAS*, *CBL*, *WT1*, *PHF6*
PV	*TET2* ****, *DNMT3A*, *ASXL1* ^§^	*JAK2* V617F, *JAK2 exon 12*	*TP53*, *RUNX1*, *SRSF2*, *U2AF1*, *IDH1/2*, *CBL*, *EZH2*, *FLT3*, *N/KRAS*, *NPM1*, *ETV6*, *SETBP1* [13,51,53,54]
ET	*TET2*, *DNMT3A*, *ASXL1*	*JAK2* V617F, *CALR*, *MPL*	
PMF	*TET2*, *DNMT3A*, *ASXL1*	*JAK2* V617F, *CALR*, *MPL*	
CMML [55,56,57,58]	*TET2*, *SRSF2*, *ASXL1*, *DNMT3A*^§§^	*TET2*, SRSF2, *ASXL1*, *N/KRAS*, *CBL*, *SETBP1*, *EZH2*	*RUNX1*, *N/KRAS*, *CBL*, *EZH2*, *U2AF1*, *SETBP1*^§§§^
CML	*TET2*, *DNMT3A*, *ASXL1*	*BCR-ABL1*	*BCR-ABL1* mutations, *ASXL1*, *IKZF1*, *TP53*, *RUNX1*, *SETD1B*
AML [59,60]	*DNMT3A*, *TET2*, *ASXL1*, *IDH1/2* *	*PML-RARA*, *MYH11-CBFB*, *RUNX1-RUNX1T1*, *MLLT3-KMT2A*, *DEK-NUP214*, *RUNX1, NPM1*, *CEBPA*, *GATA2*	*FLT3*, *N/KRAS*, *KIT*, *PTPN11*, *TP53*, *PHF6*, *SRSF2*, *STAG2*, EZH2, *RAD21*
AdvSM/MCL [38]	*TET2*, *ASXL1*, *SRSF2*	*KIT* D816V/H/Y	*RUNX1*, *CBL*, *N/KRAS*, *SRSF2*, *IDH1/2*

* Although *IDH1/2* mutations are associated with an unfavorable clinical outcome in myelodysplastic syndrome (MDS), they appear to be early events in the clonal evolution in MDS and acute myeloid leukemia (AML). In MPN, *IDH1/2* mutations usually appear as late events leading to leukemic transformation. Of note is that these mutations have not been identified in the context of CHIP/ARCH [19,61]. ** Although the *SF3B1* mutation shows a clear association with the presence of ring sideroblasts in MDS, other mutations are not specific for MDS and appear at various frequencies across other myeloid malignancies. In MDS, they represent most frequently mutated genes and are usually detectable in the founding clone. These mutations are listed here as driver mutations, but not under CHIP-like mutations, because in the context of unexplained cytopenia, their presence is highly predictive of development of a myeloid neoplasm within 5 years (95%) [62]. *** *ASXL1* gene mutations are commonly found in MDS patients. As in other myeloid malignancies these mutations are associated with an unfavorable outcome. Although they are found at similar frequencies in MDS and post-MDS sAML, they are more often sub-clonal mutations and were therefore marked here as late events [50]. **** *TET2* mutations can both precede and follow the acquisition of *JAK2* V617F in MPN. Ortmann et al. postulated that the order of acquisition of *JAK2* and *TET2* mutations has an effect on the phenotype, and that patients who acquire *JAK2* V617F mutation first and *TET2* mutation at a later time point are more likely to present with PV and have an increased risk of thrombosis [63]. ^§^ *ASXL1* mutations can occur as early events, following the acquisition of *JAK2* V617F/*CALR* mutations in MPN or as separate clones in MPN as demonstrated by Lundberg et al. [13]. They were found at higher frequency in post-PV and post-ET myelofibrosis, indicating their role in disease progression in MPN. ^§§^ These four mutations were described by Patel et al. as ancestral events in the clonal evolution of CMML [56]. All of them, except *DNMT3A,* can also appear in sub-clones, indicating that they can also be late events in the clonal evolution of CMML. Some authors consider *TET2-* and *ASXL1* mutations to be driver mutations in CMML due to their high frequency among the reported cases, and in particular the combination of *TET2* and *SRSF2* mutations which is highly prevalent in CMML. ^§§§^ Despite many articles describing the genetic basis of CMML, no mutation was clearly associated with disease progression. *RUNX1* is more frequently detected in post-CMML sAML than in CMML, however due to its high frequency in CMML the difference was not statistically significant [55]. Abbreviations: MDS, myelodysplastic syndromes; PV, polycythemia vera; ET, essential thrombocythemia; PMF, primary myelofibrosis; CMML, chronic myelomonocytic leukemia; CML, chronic myeloid leukemia; MPN, myeloproliferative neoplasms; AdvSM, advanced systemic mastocytosis; MCL, mast cell leukemia; CHIP, clonal hematopoiesis of indeterminate potential; ARCH, age-related clonal hematopoiesis.

**Table 3:** Somatic mutations producing clonal hematopoiesis of oncogenic potential (CHOP).

	Effects of the Mutant on Clonal Cells			Affected
Mutation	Differentiation	Proliferation	Oncogenesis	Myeloid Neoplasm
*BCR-ABL1_p210_*	+	+	+ *	Ph+ CML
*JAK2* V617F	+	+/-	-	MPN
*CALR* mutations	+	+/-	-	MPN
*MPL* mutations	++	+/-	-	
*KIT* D816V	++	+/-	-	ISM and AdvSM
*FIP1L1-PDGFRA*	+	+/-	-	CEL, MPN-eo
*RUNX1- RUNX1T1*	+/-	++	+	AML
*CBFβ-MYH11*	+/-	++	+	AML
*FLT3 ITD* mutations	+/-	+	+/-	AML
*NPM1* mutations	-	++	+/-	AML
*KRAS*, *HRAS* mutations	-	++	+	AML
*TP53* mutations	-	+	+	MPN, CMML, AML

* The oncogenic potential of *BCR-ABL1* is well documented and correlates with the invariable transition of (untreated) chronic phase CML into accelerated and blast phase CML. Abbreviations: Ph+ CML, Philadelphia chromosome-positive chronic myeloid leukemia; MPN, myeloproliferative neoplasms; ISM, indolent systemic mastocytosis; AdvSM, advanced systemic mastocytosis; CEL, chronic eosinophilic leukemia; MPN-eo, MPN with eosinophilia; AML, acute myeloid leukemia; CMML, chronic myelomonocytic leukemia.
